# Substrate Stiffness Modulates TGF-β Activation and ECM-Associated Gene Expression in Fibroblasts

**DOI:** 10.3390/bioengineering10090998

**Published:** 2023-08-23

**Authors:** Brijesh Kumar Verma, Aritra Chatterjee, Paturu Kondaiah, Namrata Gundiah

**Affiliations:** 1 Centre for Biosystems Science and Engineering, Indian Institute of Science, Bengaluru 560012, India; 2 Department of Developmental Biology and Genetics, Indian Institute of Science, Bengaluru 560012, India; 3 Department of Mechanical Engineering, Indian Institute of Science, Bengaluru 560012, India

**Keywords:** polydimethylsiloxane, cell–matrix interactions, collagen, MMP, TGF-β, substrate stiffness

## Abstract

Transforming growth factor-β (TGF-β) is a multifunctional cytokine that regulates the expression of ECM-associated genes during early injury. Tissue fibrosis development is driven by synergistic cues between the evolving biochemical and mechanical milieu. Few studies have addressed the role of substrate stiffness on TGF-β activity and extracellular matrix (ECM)-associated genes. We used a commercial formulation of polydimethylsiloxane (PDMS) to fabricate substrates of 40 kPa, 300 kPa, and 1.5 MPa stiffness, and cultured the HMF3S fibroblasts on substrates. We quantified TGF-β protein secreted by HMF3S cells on different substrates using a TGF-β responsive promoter reporter assay. We also tested for variations in gene expression levels on the substrates using RT-PCR and Western blotting and determined the MMP-2 and MMP-9 activities with gelatin zymography. The results showed that TGF-β protein activation was significantly compromised at lower stiffnesses. The expression of integrin α5 decreased on lower stiffness substrates and correlated with inefficient TGF-β protein activation. Collagen I, collagen III, and MMP-2 expression levels were lower on softer substrates; there was little MMP-9 activity on all substrates. Cell and nuclear morphologies were more rounded on compliant substrates, correlating with increased tubulin expression. Proliferations were higher on stiffer substrates, whereas cells on softer substrates showed cell cycle arrest. These results demonstrated critical feedback mechanisms between substrate stiffness and ECM regulation by fibroblasts, relevant in fibrosis.

## 1. Introduction

Tissue fibrosis is a result of chronic inflammation, initiated due to the immune response and accompanied with the recruitment of growth factors, proteolytic enzymes, cytokines, and angiogenic factors secreted by transformed fibroblasts that progressively remodel the affected extracellular matrix (ECM) [1,2]. Fibrosis occurs when the synthesis of de novo collagen by fibroblasts exceeds the rate at which the matrix gets degraded by metalloproteinases (MMPs) and is a primary reason underlying the dysfunction of tissues, for example, the myocardium after infarction and aneurysm growth [1,3]. Differential ECM protein expressions, cross-linking, and protease activities result in dramatic changes to tissue stiffness during fibrosis. These occur due to variations in the biophysical milieu that alter the physical properties of the ECM and biochemically influence signaling through the release of small bioactive peptides and growth factors [3,4].

Aberrant cascades of cytokines, initiated by Transforming Growth Factor- β (TGF-β), are implicated in pathologies such as wound healing, angiogenesis, and cancer [5,6]. TGF-β is secreted as a latent complex consisting of latent TGF-β binding proteins (LTBPs), Latency Associated peptides (LAPs), and mature TGF-β. Cleavage of the LAP or interactions of LTBP/LAP with integrins and other ligands releases the mature TGF-β, which can bind to the receptors and modulate the expression of ECM associated proteins such as collagen and MMPs [6,7]. ECM turnover is regulated by complex and intricate feedback processes between the cellular tension and the hierarchical assembly and organization of the substrate during remodeling [8,9]. Heterodimeric, transmembrane integrins, linking the internal cytoskeleton of cells to the ECM, sense the changing composition and stiffness and also participate in the deposition of proteins, including thrombospondin and Type I collagen [9].

Collagens perform the primary function of load bearing in tissues, provide strength to tissues, and help transfer loads to the attached cells reversibly during deformation. ECM moduli are highly variable across different organs and range from ~100 Pa in the brain and fat tissues to ~10 kPa for muscle and orders of magnitude higher in arteries and cartilage [10]. Substrate stiffness is a critical regulator of fibroblast morphology; cell stiffness is also tuned to the underlying substrate stiffness [10]. Embryonic stem cells, cultured on polydimethylsiloxane (PDMS) substrates of varied degrees of stiffness, show varied differentiation, spreading, and proliferation [11]. Earlier studies show that small-scale changes to the substrate stiffness regulate the collagen expression in cells [12,13]. Substrate stiffness can also drive changes in cellular phenotypes and promote epithelial to mesenchymal transition (EMT), which results in enhanced cell proliferation. Such changes result in the metastasis of HepG2 cells through the NEAT1/WNT/β-catenin pathway in cases of liver cancers [14]. Collagen hydrogel networks, comprising thick fibers, large pores, and higher moduli, promote mechano-signaling in stromal cells and their consequent differentiations into myofibroblasts [15]. Few recent studies demonstrate that increased ECM stiffness modulates the latent TGF-β1 activation in hepatocellular carcinoma cells [16]. How does TGF-β and the associated downstream effectors influence cellular mechanobiology on substrates of varying degrees of stiffness is a topic of intense research.

Biomaterials are useful to delineate the role of specific substrate factors that regulate the complex landscapes of cell–substrate interactions. Commonly used materials for mechanotransduction studies include biologically derived scaffolds, such as Matrigel, collagen I, fibrin, and synthetic polymers such as polyacrylamide and gelatin methacrylate gels (GelMA) [17,18]. Natural biomaterials are attractive because they reproduce the in vivo substrate characteristics but present challenges due to biological variability, degradability, variability in pore sizes, and other factors that make data interpretation difficult. In contrast, synthetic biomaterials, with surfaces modified to provide motifs for cell attachment, are attractive, as they can be repeatably tuned and permit control of mechanical characteristics such as modulus, surface topography, and pore size. Fibroblasts are activated by TGF-β in most pathophysiological conditions, including cancers, which lead to ECM remodeling. Active TGF-β secreted by cells is hypothesized to modulate the effects of stiffness changes. We tested the effect of substrate stiffness on TGF-β protein activity secreted by human mammary fibroblasts (HMF3S) using a well-characterized soft, biocompatible, and tunable PDMS elastomer. We selected substrate moduli to broadly represent collagenous matrix/muscle tissue (~40 kPa), collagenous bone (~300 kPa), cartilage (~1500 kPa), and bone (~10^7^ kPa), respectively [19]. We explored changes in integrin α5, collagen (types I and III), and MMP (2 and 9) expressions on these substrates and investigated the dependences of cell proliferations on substrates with varied degrees of stiffness. Our results demonstrated the regulation of TGF-β activity by substrate stiffness and the feedback mechanisms between cells with the underlying substrate. Most current treatments for fibrotic diseases are aimed at targeting inflammatory responses to slow the progression of fibrosis or partially reverse effects to restore homeostasis [20]. Investigations into the mechanobiology of fibroblast interactions with the substrate are essential to characterize the signaling pathways that drive MMP secretion and collagen deposition during fibrosis.

## 2. Materials and Methods

### 2.1. Cell Lines

HMF3S cells were a generous gift from Dr. P. Jat at the Ludwig Cancer Institute, UK. HMF3S, HT1080 (fibrosarcoma), and CCL-64 PAI cells were propagated in DMEM containing 10% fetal calf serum and 1% PenStrep (100 I.U/mL of penicillin and streptomycin antibiotics) in a 95% humidified CO_2_ incubator maintained at 37 °C in the study.

### 2.2. Preparation of PDMS Substrates with Various Substrate Stiffness for Cell Culture

Glass coverslips were cleaned by ultrasonication in sterile distilled water, 1 mM EDTA, 70% ethanol, and 100% ethanol for 15 min each, and air dried. Substrates were prepared by mixing silicone elastomers (Sylgard^®^ 184, Dow Corning, Midland, MI, USA) using the cross-linker in weight ratios of 10:1 (1.58 ± 0.188 MPa), 20:1 (351 ± 24 kPa), and 40:1 (41.89 ± 4.09 kPa) [18]. The solution was degassed to remove bubbles, the mixture was spin-coated on cleaned coverslips at 300 rpm for 2 min and cured in an oven at 80 °C for 2 h. The thickness of the spin-coated scaffolds were approximately 200 µm in thickness. Previous studies by Buxboim et al. [21] showed that the sensitivity of cellular responses to matrix thickness saturated beyond 20 μm thickness, with a characteristic thickness resolution of about ~5 μm. The elastic moduli of the control tissue culture dishes were approximately around 1 GPa [22]. Crosslinked PDMS substrates were plasma treated for 2 min and incubated with 40 μg/mL of fibronectin for 1 h at 37 °C to ensure uniform fibronectin coating on the surfaces (Figure S3). Substrates were washed twice with PBS, and HMF3S cells were cultured at 8000–10,000 cells/cm^2^ density.

### 2.3. RNA Isolation and Real-Time PCR

RNA was isolated using TRI^TM^ reagent (Sigma-Aldrich, St. Louis, MO, USA) based on protocols suggested by the manufacturer. Briefly, cells were washed in Phosphate Buffered Saline (PBS; pH 7.0), lysed using TRI reagent at room temperature, and chloroform was added to the mixture to separate the solution phase. The solution was centrifuged at 12,000× *g* for 10 min, and RNA was precipitated in the aqueous phase using 50% isopropanol. The resultant was suspended in 70% ethanol, and the RNA dissolved in 20 μL RNase-free water. The purity and integrity of RNA were tested using the absorbance ratio at 260 and 280 nm and formaldehyde agarose gels. A cDNA synthesis kit (Applied Biosystems, Woburn, MA, USA) was used to reverse transcribe 2 μg RNA. Gene-specific primers were used to test the expression levels of various proteins of interest. GAPDH (Glyceraldehyde 3 phosphate Dehydrogenase) was used as a normalizing control. A list of primers used in this study is included as Table S1.

### 2.4. CCL64 Assay

CCL64 is a standard assay to monitor the activity of TGF-β in condition media. The promoter of PAI (plasminogen activator inhibitor) gene fused with luciferase reporter in engineered CCL64 cells. PAI is a transcriptional target of TGF-β. As previously mentioned, [23] CCL64 cells are highly sensitive to TGF-β, making it an ideal tool to assay the minute amount of TGF-β in condition media. In the present study, we measured the active TGF-β from condition media of cells cultured on different substrate stiffness. First, we generated a standard curve using the commercially available recombinant TGF-β. Furthermore, condition media collected from cells were cultured on different degrees of stiffness. Collected media was divided into two equal halves. One half was left on ice as it is, and the other half was heated at 85 °C for 10 min to fully activate TGF-β protein. Following this, CCL64-PAI cells were treated with these two-condition media, and luciferase activity was measured using dual luciferase assay system (Promega) from these cells. Finally, these readings were used to extrapolate concentration from the standard curve.

### 2.5. Western Blot Asaay

For Western blot assay, cells were washed with PBS and lysed in RIPA (Radio-immunoprecipitation assay) buffer containing 50 mM Tris-HCl buffer (pH 7.4), 1% NP-40, 0.25% deoxycholate, 150 mM NaCl, 1 mM EDTA (pH 8.0), 1 mM PMSF, 1 mM NaF, 1 mM sodium orthovanadate, 1× protease inhibitor cocktail III (stock 100×, Calbiochem, San Diego, CA, USA), and 0.1% SDS. Lysates were stored on ice for 10–15 min and centrifuged for 10 min at 12,000× *g* and at 4 °C. A total of 20 μg of total cell lysate was resolved by 12.5% SDS-PAGE and blotted onto immobilon membranes by electroblotting. Antibodies used in this study were pFAK Tyr397 (#3283S, CST, Denver, CO, USA), FAK (#3285, CST, USA), ITGA5 (#ITT05200, GTI, Trinity Drive Stafford, TX, USA), tubulin-α (#ITT4777H, GTI, Trinity Drive Stafford, TX, USA), β-actin (#ITM2093, GTI, Trinity Drive Stafford, TX, USA), and GAPDH (#3683S, CST, Denver, CO, USA).

### 2.6. Immunocytochemistry

Cells were fixed using 3.7% paraformaldehyde (PFA) for 25 min and blocked with 10% FBS at room temperature for 1 h. To visualize the actin cytoskeleton, cells were incubated with rhodamine-phalloidin (Thermofisher, Waltham, MA, USA, 1:200) for 60 min. Hoechst (Thermofisher, 1:400; 15–20 min) was used to stain the nucleus. Samples were imaged using a confocal microscope (ZEISS LSM 900 with Airyscan 2) at 10× and 63× magnifications, respectively.

### 2.7. Zymography

Zymography was performed to examine the enzymatic activity of matrix metalloproteinases (MMP-2 and MMP-9) in the conditioned media of HMF3S cells. Cells were seeded on PDMS substrates, and the conditioned medium was collected and centrifuged (5000× *g*) to pellet the cell debris. Zymography was performed as previously described [24]. Briefly, the spent medium was transferred to a fresh tube, and 20 µL of conditioned media was loaded on a 12.5% SDS-PAGE gel with 0.5% gelatin for electrophoresis. In addition to the SDS-PAGE–gelatin gel, the conditioned media was also resolved on one normal 12.5% SDS-PAGE. Normal gel was used as a loading control. The resulting gels were incubated in Triton × 100 (2.5%) for 30–40 min and placed overnight in an incubation buffer (Tris- HCl buffer pH 7.5–10 mM, Triton × 100–1.25%, CaCl_2_-5 mM, ZnCl_2_-1 µM; 37 °C) to quantify enzymatic activity. Coomassie brilliant blue (15–20 min at room temperature) was used to stain the gels. Images of the de-stained gel obtained using methanol (40%), glacial acetic (10%), and water (50%) were acquired using a Chemidoc imaging system (ChemiDoc XRS and Gel Imaging System).

### 2.8. Cell Cycle Analysis

HMF3S cells were seeded on substrates for 24 h, washed with chilled PBS, and lysed for 30 min using an iced hypotonic solution containing propidium iodide (PI), sodium citrate, Triton X −100, and double-distilled water. The cell lysate was centrifuged at 600× *g* for 5 min at 4 °C, the supernatant discarded, and the pellet containing the nuclei was resuspended in a hypotonic solution containing propidium iodide (PI). Samples were analyzed using FACS (BD Accuri C6).

### 2.9. Trypan Blue Exclusion Assay

Cells were cultured in PDMS-coated coverslips kept in 35 mm dishes for 24 h. Following this, they were trypsinized and incubated in 0.4% trypan blue solution for 3 min at room temperature. Live and dead cells were counted using a hemocytometer.

### 2.10. Measurement of Cell and Nuclear Morphometric and Densitometric Analysis

The cell and nuclear aspect ratios were calculated for single cells using ImageJ (NIH) [25]. Confocal images of single cells were segmented to identify the boundaries, and the corresponding areas were obtained from binarized images. The nucleus was fit to an ellipse, and the aspect ratio was determined as a ratio of the major to the minor axis [26]. Western blot densitometric analysis was performed using ImageJ as previously described [25].

### 2.11. Statistical Analysis

Statistical differences in the mean values between groups, using three biological repeats, were obtained using ANOVA with Bonferroni multiple comparison tests in GraphPad Prism (v6.0, GraphPad Software, Inc., La Jolla, CA, USA). *p*-values < 0.05 were considered statistically significant.

## 3. Results and Discussion

### 3.1. Substrate Stiffness Regulates TGF-β Activation

**Figure 1:**
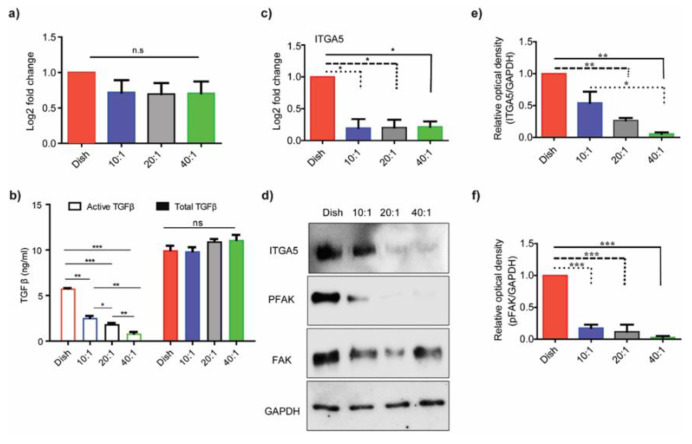
**Substrate stiffness regulates TGF-β activity and the expression of integrin α5.** (**a**,**c**) RNA collected from cells seeded on petri dish; 10:1 (1.58 MPa), 20:1 (351 kPa), and 40:1 (41.89 kPa) PDMS substrates were analyzed for TGF-β1 (**a**) and integrin α5 mRNA expression (**c**). (**b**) TGF-β concentration from condition media quantified using CCL64-PAI assay. (**d**) Immunoblots of integrin α5, FAK, and pFAK from cell lysates collected from cells seeded on dishes 10:1 (1.58 MPa), 20:1 (351 kPa), and 40:1 (41.89 kPa) PDMS substrates and their densitometric quantifications (**e**,**f**). All experiments were repeated in triplicates in the reported analysis. Data are represented as mean ± SEM; * *p* < 0.05; ** *p* < 0.01; *** *p* < 0.001.

Fibrosis is the stiffening of the tissue that happens due to dysregulated expression of ECM due to chemokines, such as TGF-β, that may influence the fibroblast mechanobiology in tissues. A recent study by Chakravarthy et al. also investigated the correlations between ECM genes upregulated in cancer and TGF-β signaling in cancer-associated fibroblasts [20]. We explored possible modulations of TGF-β expression in HMF3s cells on PDMS substrates of varying degrees of stiffness. The mRNA expression of TGF-β, obtained using qRT-PCR (Figure 1a), showed marginal reductions at the transcript level in cells on PDMS substrates compared to control dishes. These differences were, however, not statistically significant. To explore the effect of substrate stiffness on TGF-β protein activation, we used the CCL64-PAI assay to quantify TGF-β protein in the media. In this method, we use the promoter of the PAI (plasminogen activator inhibitor) gene, a transcriptional target of TGF-β, fused with the luciferase reporter, engineered in CCL64 cells to assay TGF-β. We measured the active TGF-β content in the conditioned media collected from cells cultured on substrates of different degrees of stiffness. We generated the TGF-β response standard curve using commercially available recombinant TGF-β protein (Figure S1) and divided the serum-free condition media, collected from conditioned media from cells on different substrates, into two parts. The first was heated (85 °C; 10 min) to activate TGF-β (active), and the second was kept on ice (native). CCL64-PAI cells were treated with the two conditioned media separately. The comparisons showed no apparent differences in the heat-activated condition media from HMF3S cells on the different PDMS substrates. In contrast, there was a gradual decrease in the activity of native condition media with decreasing stiffness (Figure 1b). The results demonstrated that there were no significant changes in the total TGF-β expression either at the intracellular or extracellular levels; the active TGF-β content in the conditioned media, however, changed with the substrate’s stiffness.

Substrates of higher stiffness are essential for the activation of fibroblasts, through TGF-β, which results in higher cell contractility and matrix remodeling [27,28]. Activation of fibroblasts into myofibroblasts alters the ECM gene expression, regulated by TGF-β [28]. TGF-β is secreted as a latent complex, consisting of latent TGF-β binding protein (LTBP), latency-associated peptide (LAP), and mature TGF-β. Activation of the mature TGF-β dimer, through proteolytic cleavage of LAP and/or interaction of LTBP with integrins, is essential for receptor binding. Fibroblasts hence regulate the activation of TGF-β, based on the underlying substrate stiffness. Integrins promote the spreading of cells on substrates and participate in signaling through cross-talk between the substrate ligands and the cell’s cytoskeleton. We tested the effect of substrate stiffness on integrin α5 expression in HMF3S cells cultured on PDMS substrates of different degrees of stiffness and used the culture dish as a control. Integrin α5 domains in fibroblasts selectively attach through fibrillar adhesions to fibronectin ligands on the substrate. These interactions change the integrins from low- to high-affinity conformations, anchoring the cell to the substrate [29]. Signaling adaptors of integrins include kinases, like FAK, which are highly dynamic and recruit other molecules to organize the cytoskeleton [29]. We quantified changes in the expression levels of integrin α5 (ITGA5), FAK, and phospho-FAK to test for differences in cell signaling on PDMS substrates of varying degrees of stiffness. Figure 1c shows a significant reduction in ITGA5 at the transcript level on PDMS substrates 20:1 (351 kPa) and 40:1 (41.89 kPa) as compared to the dish (*p* < 0.01). We next compared fold changes in ITGA5 values and show clear differences between the control and PDMS substrates (Figure 1d). The relative optical densities of ITGA5 decreased on the PDMS substrates as compared to controls (Figure 1e). Differences in the relative optical densities of ITGA5 on the PDMS substrates were, however, discernible only between the 10:1 (1.58 MPa) and 40:1 (41.89 kPa) PDMS substrates (Figure 1e; *p* < 0.05). FAK tyr397 phosphorylation showed a direct correlation with the integrin α5 protein expression on the substrates (Figure 1f). Our observations agreed with previous studies by Gimenez et al., who demonstrated that TGF-β1 activity correlated with the enhanced activation of the FAK/Akt pathway in fibroblast cells extracted from idiopathic pulmonary fibrosis (IPF) patients [30]. These results suggested that changes to substrate stiffness regulate the integrin α5 expression on PDMS substrates, alter signaling, and corroborate an earlier study by Yeh et al., which showed that β1 integrin expression was regulated by matrix stiffness [22]. Mechanically induced changes to the integrin conformation may hence help in mechanotransduction to sense the substrate’s characteristics.

### 3.2. ECM-Associated Genes Are Downregulated at Lower Degrees of Stiffness

**Figure 2:**
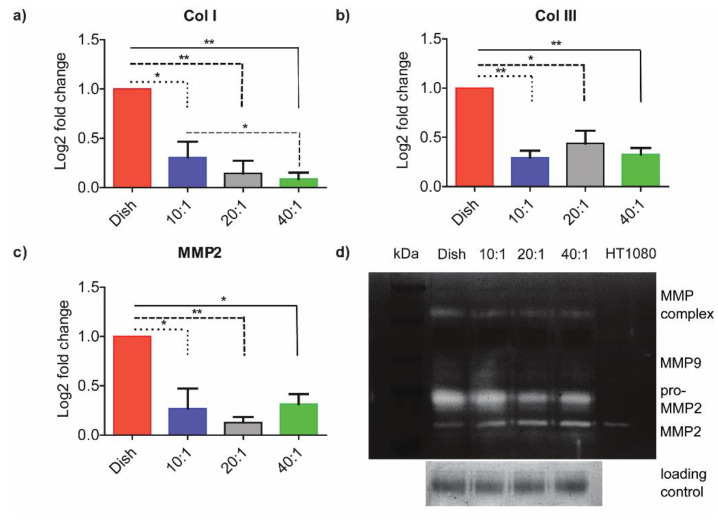
**Variations in ECM-associated gene expression and activity varies with substrate stiffness**. qRT-PCR expression analysis of (**a**) Col I, (**b**) Col III, and (**c**) MMP2 are shown from RNA collected from cells cultured on dish, 10:1 (1.58 MPa), 20:1 (351 kPa), and 40:1 (41.89 kPa) substrates, respectively. (**d**) Gelatin zymography with condition media collected from cells cultured on dish, 10:1 (1.58 MPa), 20:1 (351 kPa), and 40:1 (41.89 kPa) PDMS substrates were assayed for MMP-2 and MMP-9. All experiments were performed in biological triplicates. Data are represented as mean ± SEM; * *p* < 0.05; ** *p* < 0.01.

We quantified differences in the expression levels of collagen I, collagen III, and MMPs on the PDMS substrates of differential degrees of stiffness and compared results from these experiments using fibroblasts on control dishes. Figure 2a shows that the ColI expression gradually decreased with stiffness. The results showed a statistically significant difference between the 10:1 PDMS (1.58 MPa) and 40:1 PDMS (41.89 KPa) group. There were also significant differences between the control and prepared PDMS substrates of different degrees of stiffness. We used Real-time PCR to analyze the expressions of collagen I, collagen III, and MMP genes in cells on substrates with different degrees of stiffness. The expression levels of these genes were significantly lower on compliant substrates as compared to stiffer ones (Figure 2a–c). These observations agreed with earlier studies by Gimenez et al., who showed that TGF-β1 consistently increased COL1A1 mRNA levels on stiffer matrices [30]. We hypothesized that substrate stiffness may also affect MMP activity. We used gelatin zymography to assess the effects of substrates on MMP-2 and MMP-9 activities (Figure 2d). Conditioned media from cells on different degrees of substrate stiffness were resolved on 12.5% of SDS-PAGE containing 5% gelatin, and the gel was transferred to the MMP activation buffer. Regions of gelatin that degraded due to MMP activity did not stain with Coomassie Blue. Results from HT1080 cell condition media, containing both MMP-2 and MMP-9, were used as a positive control. Gelatin zymography showed little MMP-9 activity or any significant change in MMP-9 transcript level on substrates of differing stiffness (Figure 2d). A quantification of Figure 2d is provided in supplementary Figure S2. The conversion of MMP-2 to active-MMP-2 was significantly higher on lower stiffness substrates as compared to stiffer ones. Our results clearly demonstrated that HMF3S fibroblasts altered their matrix production (Col I and Col III) and MMP secretions based on substrate stiffness.

Cells deposit, crosslink, and break down the surrounding ECM using microenvironment cues. Changes to matrix stiffness are hypothesized to trigger pathways for increased ECM deposition by activated fibroblasts, characterized by the expression of α-smooth muscle actin (α-SMA), which are essential in fibrosis [22,28,29,31,32,33,34,35]. Lachowski and co-workers showed that the modulus of polyacrylamide substrates modulated gene expressions of MMP-2, MMP-9, and TIMP-1 in hepatic stellate cells [29]. The moduli of substrates investigated in their study varied from 4 kPa to 25 kPa, which was significantly lower as compared our study. Transcriptional activation by YAP/TAZ, coupled with higher substrate stiffness, triggered its localization to the nucleus, which was accompanied with increased matrix production [28,36]. The deposited matrix may subsequently undergo crosslinking and also be associated with altered MMP regulation. MMPs are the primary enzymes linked to matrix remodeling that are associated with cancer cell migration and metastasis [36]. Increased upregulation of MMP activity in pancreatic cells, in response to substrate stiffness, is mediated through higher cell contractility [37]. Our results suggest that the feedback between stiffening and degradation of the ECM by HMF3S cells may hence promote early fibrosis in tissues.

### 3.3. Substrate Stiffness Modulates Fibroblast Morphology and Proliferation

**Figure 3:**
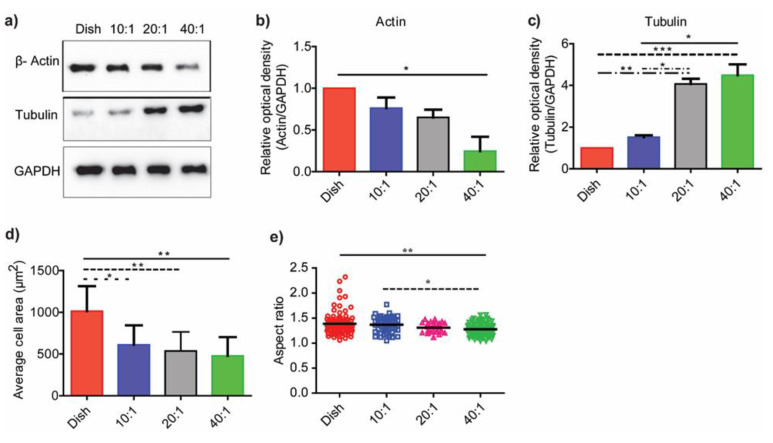
**Substrate stiffness regulates the cytoskeletal gene expression**. (**a**) Immunoblot of β-actin and tubulin from cell lysate collected from cell seeded on dish, 10:1 (1.58 MPa), 20:1 (351 kPa) and 40:1 (41.89 kPa) substrate. Densitometric quantitation results using ImageJ were obtained for (**b**) β-actin and (**c**) tubulin using the immunoblots. (**d**) Average fibroblast areas on substrates of varied degrees of stiffness were obtained using the confocal images (N~20 for each group). (**e**) Aspect ratios of cells on substrates of varied degrees of stiffness were obtained using analysis from the confocal image of nucleus (N~40 for each group). All experiments were performed in biological triplicates. Data are represented as mean ± SEM; * *p* < 0.05; ** *p* < 0.01; *** *p* < 0.001.

We used PDMS of various stiffness ratios and quantified the expression levels of cytoskeletal proteins, actin and tubulin, on the various substrates (Figure 3a). We compared the optical densities of proteins on the various substrates relative to GAPDH levels (*n* = 3). Figure 3b shows that actin decreased on substrates with lower stiffness; significant results were discernible between control and the 40:1 (41.89 kPa) PDMS substrates alone (*p* < 0.05). In contrast, tubulin significantly increased with a decrease in substrate stiffness (Figure 3c). The differences in tubulin expression levels were apparent on the 20:1 (351 kPa) and 40:1 (41.89 kPa) PDMS substrates as compared to the other substrate stiffnesses in this study.

**Figure 4:**
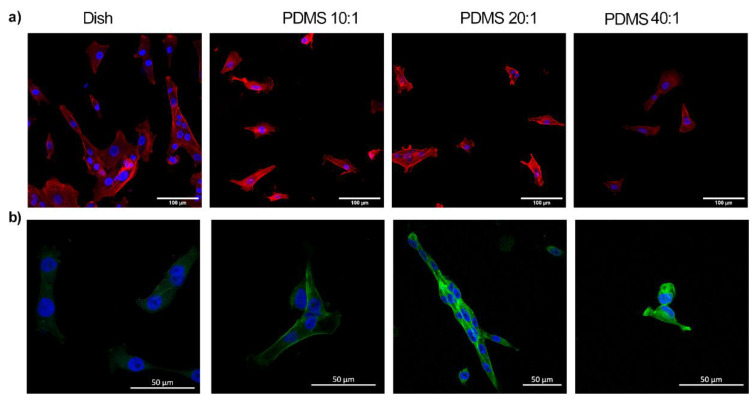
Confocal images of cells seeded on Petri dish; 10:1 (1.58 MPa), 20:1 (351 kPa), and 40:1 (41.89 kPa) PDMS substrates show differences in the cell morphologies. (**a**) The upper panel is stained for actin (red), using phalloidin red, and DAPI, which stains the nuclei blue. Scale bar: 100 μm. (**b**) The lower panel shows tubulin (green) and DAPI-stained cells (blue). All experiments were performed in biological triplicates. The scale bar is 50 μm.

Poly(dimethylsiloxane) has many properties, including gas permeability, biocompatibility, low water absorption, ease of microfabrication, high stability, and mechanical property tuneability, which make it a favorable biomaterial for mechanobiology experiments [38,39,40]. The inherent hydrophobicity of PDMS dictates interactions with biological samples that adhere to the surface; there are, however, few differences in the amount of adsorbed protein to the substrates [41,42]. Plasma treatment of PDMS results in a hydrophilic and rough surface through surface oxidization [43]. Cell adhesions to substrates are modulated by factors such as ligand type and substrate stiffness [22]. Our results show correlations between changes in cell morphologies with the underlying substrate stiffness. The adherent fibroblast areas on PDMS substrates were different as compared to cells on control dishes (Figure 3d and Figure 4). The average cell spread area (*n* > 40 each group) was lower on PDMS substrates with lower stiffness as compared to control dishes (*p* < 0.01). There were, however, no differences in the cell spread areas on PDMS substrates of various stiffness. Nuclei on stiffer substrates were less rounded on control substrates as compared to those on compliant substrates. The aspect ratios of nuclei decreased with a decrease in substrate stiffness; these data clearly demonstrated a rounding of the nucleus on compliant substrates (Figure 3e).

**Figure 5:**
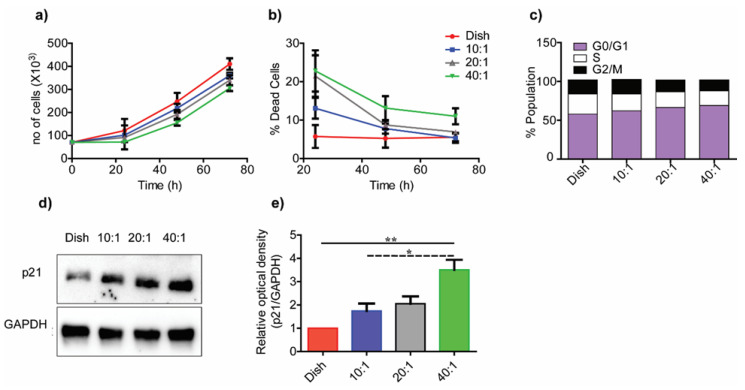
**Reduction in substrate stiffness restricts cell growth.** (**a**) Cell proliferation on Petri dish; 10:1 (1.58 MPa), 20:1 (351 kPa), and 40:1 (41.89 kPa) PDMS substrates were obtained using trypan blue assay. (**b**) The percentage of dead cells in each group are shown at 24 h, 48 h, and 72 h. (**c**) Histogram representing the percentage of cells in different cell cycle phases shown for substrates of different degrees of stiffness. (**d**) The immunoblot of p21, and (**e**) densitometric analysis of immunoblot using ImageJ. All experiments were performed in biological triplicates. Data are represented as mean ± SEM; * *p* < 0.05; ** *p* < 0.01.

As microtubules are the primary components of the mitotic spindle, we hypothesized that cell proliferation should differ on substrates of differing moduli. Figure 5a shows differences in the cell growth over a 3-day period on PDMS substrates of varied degrees of stiffness. Cellular growth on stiffer substrates over the 24 h period was significantly higher on the control substrate compared to the 40:1 (41.89 kPa) compliant substrate (*p* < 0.01). Although cells proliferated on all substrates, those seeded on higher stiffness substrates showed higher proliferation rates. We used the trypan blue live–dead cell assay to distinguish between dead and live cells on all substrates. Control substrates had <10% dead cells at 24 h in contrast to 10:1 (1.58 MPa), 20:1 (351 kPa), and 40:1 (41.89 kPa) PDMS substrates that showed over 10% cell death (Figure 5b). Dead cell percentage in the population decreased significantly at later time points. All substrates, barring the 40:1 (41.89 kPa) substrate, had <10% dead population at 72 h.

We used FACS profiling for cells cultured on substrates of different degrees of stiffness. Cells were initially synchronized through serum starvation for 24 h, and normal media was added to release the cell cycle (Figure 5c). Cells were enriched in G0/G1 cycle phase on the lower stiffness substrates, which suggested possible cell cycle arrest. The expression of p21 protein, used as a marker for cell cycle arrest, showed significantly higher expression on lower stiffness substrates compared to the control substrates (Figure 5d,e) [31]. These data agreed with others that demonstrated increased proliferation of mammary epithelial cells, vascular smooth muscle cells, and mouse embryonic fibroblasts on stiffer substrates fabricated using alginate [31,33]. Increased focal adhesion kinase (FAK) activity on stiffer substrates was linked to higher cell proliferation [34]. Studies also suggested the existence of a possible tuning mechanism in cells to substrates with stiffness similar to the target tissue from which they are derived [9]. The specific mechanisms underlying these behaviors are presently not well understood.

## 4. Conclusions

Form and function in cells are inextricably influenced by the biochemical and mechanical environment. The basement layer of tissues primarily comprised fibroblast cells that responded to changes in their micro-environment. Biochemical cues such as TGF-β influenced cell growth and the extracellular milieu. Their role in pathogenic conditions is very well elaborated. However, the role of biomechanical cues on cellular behavior have not been investigated. The role of TGF-β during pathophysiological conditions, like fibrosis and solid tumor development, is well characterized, but little is understood concerning how TGF-β expression changes with stiffness. Our study shows how physical cues, such as matrix stiffness, modulate TGF-β protein activation over a large range of substrate stiffness from 40 kPa to 1.5 MPa. Earlier studies compared changes in the mechanobiology of cells on substrates of moduli ~1–10 kPa; few studies have quantified the role of substrate stiffness on TGF-β activation on substrates of higher moduli ranging from 0.1 to 1 MPa [16,22,33]. We used PDMS substrates prepared with varying cross-linking ratios to obtain elastic moduli over a wider range of values as compared previous studies. The use of well-characterized biomaterials with tunable and biocompatible properties, such as PDMS, makes it useful in biological studies, including single cell and immunoassays, stem cell research, microarrays, and in mechanobiological studies to investigate cellular responses to the underlying matrix stiffness [42,43,44,45,46,47].

Our results demonstrated that TGF-β activity decreased with a lowering of the substrate stiffness. Integrins play an important role in TGF-β activation. The integrin expression and signaling correlated with TGF-β activity in our study. ECM-associated gene expressions for collagen I, collagen III, and MMP-2 decreased on compliant PDMS substrates compared to stiffer ones. There were, however, no significant changes at the transcript level in MMP-9 on all degrees of substrate stiffness. We used gelatin zymography to test for differences in MMP-2 and MMP-9 activity levels from cells on varied substrates. Our data showed little MMP-9 activity on all substrates and higher conversion of pro-MMP-2 to active-MMP-2 on lower stiffness substrates. These results showed the critical feedback between substrate stiffness and ECM regulation by fibroblasts. Fibroblasts had higher spread areas on control Petri dishes compared to PDMS substrates of varied degrees of stiffness. Cell nuclei were rounded on compliant substrates and correlated with increased tubulin expression levels. Cell proliferation was higher on stiffer substrates and showed cell cycle arrest on lower stiffness substrates. These studies may be extended to quantify the effects of the mechanical microenvironment on TGF-β activation in 3D scaffolds to aid an understanding of the processes that contribute to the metastatic progression of cells in solid tumors. The application of external mechanical stimuli, such as cyclic stretch or fluid shear stresses, to cells on substrates with varying degrees of stiffness may also regulate the ECM-associated gene expressions and TGF-β protein activation. Tuning of the substrate moduli with different formulations of PDMS to generate stiffer or more compliant membranes may also be useful to test the differential TGF- β protein activation response in future studies. Earlier studies have demonstrated specific mechanisms of TGF-β activation [27,28] and downstream changes in ACTA-2 expression [48], phospho-Smad2 levels [49], TGF-β induced activation of the FAK/Akt pathway [50], and other markers due to variations in substrate stiffness. Although the roles of TGF-β in cell proliferation, growth, and ECM remodeling are reported in the literature, we did not test the status of TGF-β signaling on substrates of various stiffness to delineate the primary and secondary effects of substrate stiffness on HMF3s cells. Future studies to investigate the effects of TGF-β inhibitors on cells cultured on different degrees of substrate stiffness will help establish the inter-dependence of altered ECM expression and cell proliferation due to TGF-β signaling.

## Data Availability

All data are contained within the article and supplementary materials.

## Supplementary Materials

The following are available online at https://www.mdpi.com/article/10.3390/bioengineering10090998/s1, Table S1: Primers used in the study are indicated. Figure S1: Standard curve for recombinant TGF-β protein response using CCL64 PAI assay is shown. Figure S2: Quantification of Gelatin zymography images (Figure 2d) with condition media collected from cells cultured on dish, 10:1 (1.58 MPa), 20:1 (351 kPa) and 40:1 (41.89 kPa) PDMS substrates were assayed for MMP-Complex, Pro-MMP-2 and MMP-2. Data are represented as mean ± SEM; * *p* < 0.05; ** *p* < 0.01. Figure S3: Tile scan images taken in a live cell fluorescent microscope showing uniform coating of fluorescently tagged fibronectin on PDMS substrates with different stiffness (10:1, 20:1, 40:1).
