# Supplementary material for: Substrate Stiffness Modulates TGF-β Activation and ECM-Associated Gene Expression in Fibroblasts
Source: Bioengineering (Basel). 2023 Aug 23;10(9):998. doi: 10.3390/bioengineering10090998 (PMC10525202; doi:10.3390/bioengineering10090998)
Supplement: Supplementary file 1 [file bioengineering-10-00998-s001.zip › bioengineering-2292362-supplementary.pdf]

## Supplementary Figures

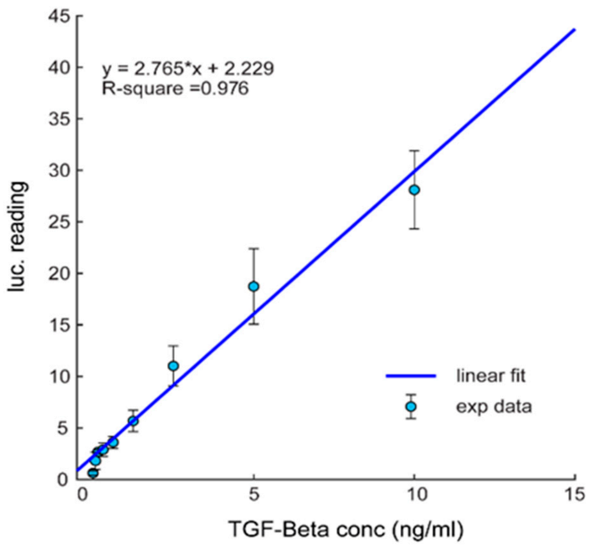


**Figure S1**: Standard curve for recombinant TGF-β protein response using CCL64 PAI assay is shown.

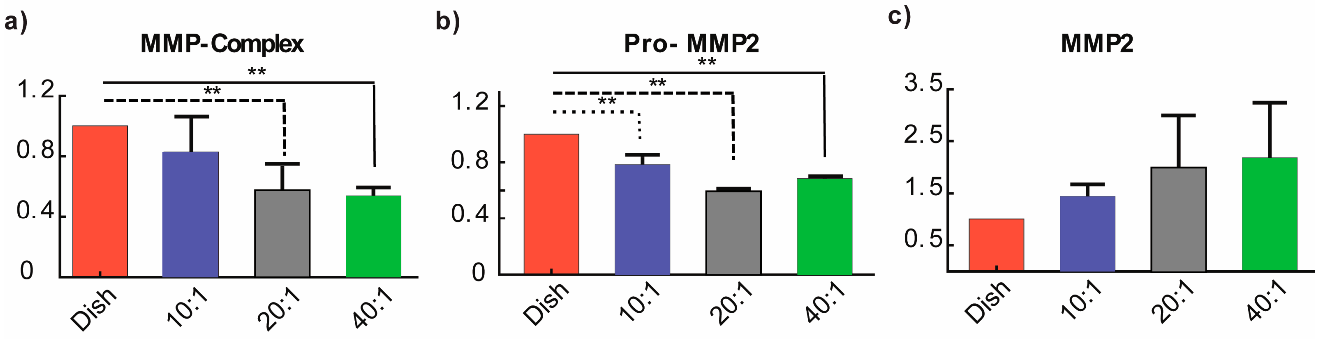


**Figure S2**: Quantification of Gelatin zymography images (Fig. 2d) with condition media collected from cells cultured on dish, 10:1 (1.58 MPa), 20:1 (351 kPa) and 40:1 (41.89 kPa) PDMS substrates were assayed for MMP-Complex, Pro-MMP-2 and MMP-2. Data are represented as mean ± SEM; * $p<0.05$; ** $p<0.01$.

**10:1** **20:1** **40:1**

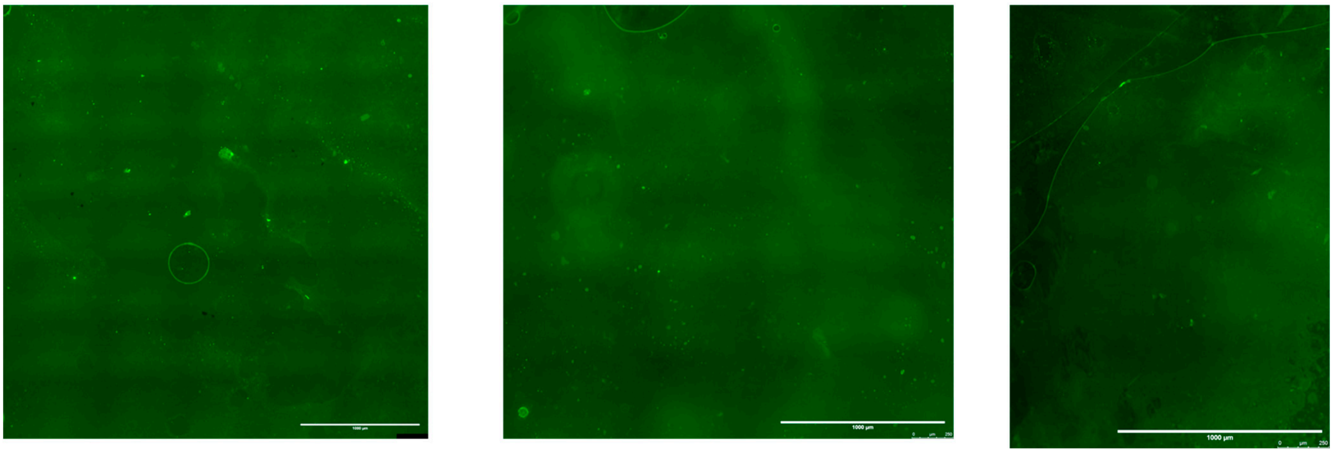

**Figure S3.** Tile scan images taken in a live cell fluorescent microscope showing uniform coating of fluorescently tagged fibronectin on PDMS substrates with different stiffness (10:1, 20:1, 40:1).

## Supplementary Table

**Table S1**: Primers used in the study are indicated.

| Gene | Sequence |
|---|---|
| **MMP2_FP** | 5’ GCGGCGGTCACAGCTACTT 3’ |
| **MMP2_RP** | 5’ CACGCTCTTCAGACTTTGGT 3’ |
| **Col I_FP** | 5’ GTGATTGGGCGGGATGTCTTCGTC 3’ |
| **Col I_RP** | 5’ CCCCAGCCACAAAGAGTCTACA 3’ |
| **Col III_FP** | 5’ TCCTGAGGACCAGTAGGCA 3’ |
| **Col III_RP** | 5’ CGCCCTCCTAATGGTCAAGG 3’ |
| **TGF-β1_FP** | 5’ TACCTGAACCCGTGTTGCTCTC 3’ |
| **TGF-β1_RP** | 5’ GTTGCTGAGGTATCGCCAGGAA 3’ |
| **GAPDH_FP** | 5’ GTCTCCTCTGACTTCAACAGCG 3’ |
| **GAPDH_RP** | 5’ ACCACCCTGTTGCTGTAGCCAA 3’ |
